# Premedication with dexmedetomidine to reduce emergence agitation: a randomized controlled trial

**DOI:** 10.1186/s12871-019-0816-5

**Published:** 2019-08-07

**Authors:** Jong Chan Kim, Jihee Kim, Hayeon Kwak, So Woon Ahn

**Affiliations:** Department of Anesthesiology and Pain Medicine, CHA Bundang Medical Center, CHA University, 59 Yatap-ro, Bundang-gu, Seongnam-si, Gyeonggi-do 13496 South Korea

**Keywords:** Dexmedetomidine, Emergence agitation, Nasal bone fracture

## Abstract

**Background:**

Nasal bone fracture is the most common type of facial fracture, and the high incidence of severe emergence agitation occurring after closed reduction of the nasal bone fracture can be challenging to manage. The purpose of this trial was to evaluate whether pre-operative administration of dexmedetomidine is effective in reducing the incidence and severity of emergence agitation in adults undergoing closed reduction of nasal bone fractures.

**Methods:**

In this randomized controlled trial, 90 patients who were scheduled to undergo closed reduction of a nasal bone fracture were prospectively included and were randomly assigned to either the control group (*n* = 45; 0.9% saline infusion) or the dexmedetomidine group (n = 45; 1 μ/kg over 10 min, pre-operatively). The primary endpoint was Aono’s four-point scale scores after anesthesia. The recovery time and numeric rating scale score were assessed as secondary endpoints.

**Results:**

Aono’s four-point scale scores were lower in the dexmedetomidine group than in the control group (median: 1 [1] vs. 1 [1, 2], 95% confidence interval of difference: 0.01 to 0.02, *P* = 0.02). The number, severity, and duration of agitation episodes were significantly lower in the dexmedetomidine group than in the control group. Furthermore, the number of patients exhibiting intraoperative movement was lower in the dexmedetomidine group.

**Conclusions:**

Pre-operative administration of dexmedetomidine demonstrated several significant benefits, such as a lower incidence of emergence agitation, reduced agitation severity, and a shorter duration of agitation. Additionally, we observed more stable maintenance of intraoperative anesthesia with less movement during the surgery.

**Trial registration:**

Identifier: KCT0000585 (registration date: 12–19- 2012).

## Background

Nasal bone fracture is the most common type of facial fracture [1] and the third most common fracture type of the human skeleton [2]. One treatment method for nasal bone fracture is closed reduction (CR), which can be performed under local or general anesthesia [3]. General anesthesia facilitates patient comfort, improves patient satisfaction, [4] and helps maintain patient immobilization during surgery. However, external splints or nasal packing is also commonly used for stabilization after CR because there is no pinning or fixation of the nasal bones. These can be uncomfortable for patients, causing difficulty with breathing; this contributes to the incidence of emergence agitation (EA), which can cause re-dislocation of a corrected fracture.

EA can necessitate physical or chemical restraint of the patients [5]. EA commonly occurs after otolaryngological procedures, which poses additional challenges [6]. The incidence of EA following nasal surgery has been reported to be 4.7–27.3% [7, 8].

Dexmedetomidine, a specific α^2^-adrenergic receptor agonist, has been shown to be effective in preventing EA in children [9–12]. According to a recent meta-analysis, intraoperative administration of dexmedetomidine decreases postoperative pain and the incidence of EA in adults [13]. The objective of the present study was to evaluate whether pre-operative intravenous infusion of dexmedetomidine is effective in reducing the incidence and severity of EA in adults undergoing CR of a nasal bone fracture.

## Methods

**Fig. 1:**
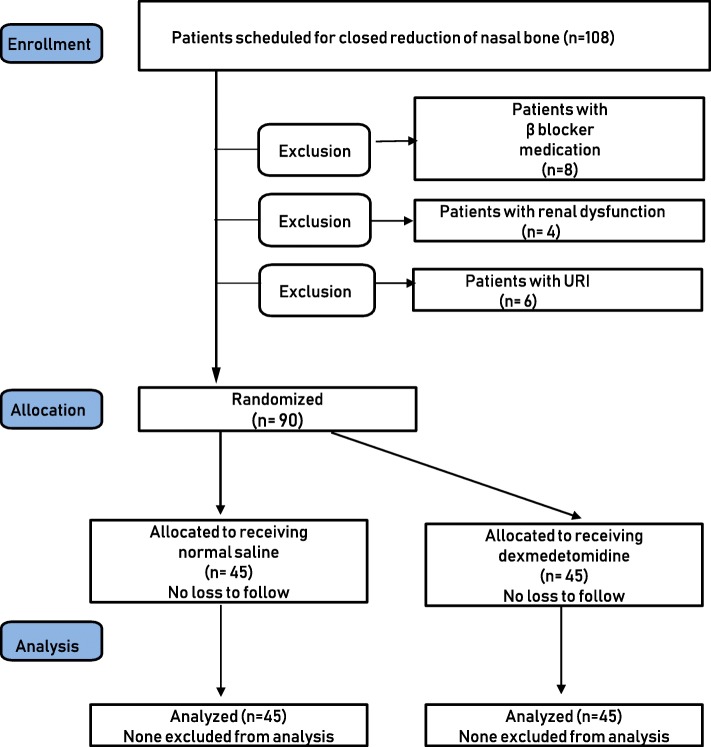
Flow chart of patient participation

After obtaining approval from the Institutional Review Board (IRB number: 12–071, protocol number: Ver 1, Date of approval: 9/26/2012), written informed consent was obtained from all enrolled participants. This manuscript adheres to the applicable CONSORT guidelines. The participants in this study were 20 to 60 years of age, American Society of Anesthesiologists class 1 or 2, and were scheduled to undergo CR of a nasal bone fracture under general anesthesia between November 2012 and September 2013. One-hundred eight patients were prospectively included. Patients were excluded from the study if they had a known history or clinical evidence of chronic obstructive pulmonary disease or respiratory insufficiency based on pre-operative medications or a pulmonary function test. Patients were also excluded if they had a history of renal or hepatic dysfunction, sleep apnea syndrome, recent symptoms of an upper respiratory infection, or if they were taking beta-blockers. Ninety patients were randomly assigned to either the control group or dexmedetomidine group one day before surgery. Randomized group allocation was performed using a computerized randomization table created by one staff member who was not involved in the patient’s anesthesia or recovery care. The flowchart of the patient enrollment process is shown in (Fig. 1). Staff members who provided clinical care for participants and collected the clinical data for this study were blinded to the patients’ group allocation.

Standardized anesthetic management was provided to all patients. Upon arrival at the pre-anesthetic room, standard monitoring was applied to each patient by an attending nurse who was not involved in this study, which included electrocardiography, pulse oximetry, and a non-invasive blood pressure cuff. Patients were re-educated that they might feel discomfort postoperatively due to the nasal packing. The control group received 0.5 ml•kg^− 1^ 0.9% saline intravenously over 10 min before anesthetic induction. The dexmedetomidine group received dexmedetomidine 1 μg•kg^− 1^ (Precedex, Hospira Worldwide, Lake Forest, IL, USA) in an equal volume of saline, intravenously, over 10 min before anesthetic induction. All the study medications were administered by a single researcher, who was not involved in any other part of this study. No other sedative premedications were given to the study participants.

On arrival at the operating theatre, we checked the patient’s sedation scale (Richmond Agitation-Sedation Scale, RASS) scores before anesthetic drug administration. Anesthesia was induced with fentanyl 1 μg•kg^− 1^ and propofol 2 mg•kg^− 1^. The patient’s lungs were ventilated using a face mask with 100% oxygen, and succinylcholine 1 mg•kg^− 1^ was administrated intravenously. After muscle relaxation was achieved, the patient’s trachea was intubated. During the operation, anesthesia was maintained with fentanyl and sevoflurane which was titrated to maintain a bispectral index (BIS) value between 40 and 60. Mechanical ventilation parameters during anesthesia were standardized to maintain normocarbia. If the patient’s blood pressure was 20% lower than the baseline value, intravenous ephedrine was administered. At the conclusion of the operation, once the nasal splint was attached, all anesthetic agents were discontinued, and 100% oxygen was administered. After confirmation that the patient had fully recovered from muscle relaxation and after the patient opened his or her eyes to verbal stimulation, the trachea was extubated, and the patient was transferred to the post-anesthesia care unit (PACU).

Because patients responded to verbal stimulation, EA was assessed using Aono’s four-point scale (Appendix), and pain was evaluated using the numeric rating scale (NRS, 0–10) at 2-min intervals until the patient was discharged from the PACU; the peak NRS and Aono’s scores were then recorded. Anesthesia time, operative time, the time from the end of the operation to extubation, and the duration of EA were recorded by the patient’s attending anesthesiologist. In the PACU, if the patient requested additional analgesics or if the patient’s NRS score was 3 or more, meperidine 25 mg was administered intravenously and repeated as required. All doses of rescue analgesics and the duration of the PACU stay were also recorded by an attending PACU staff who was not involved in the anesthesia administration or the current study.

The primary endpoint of this study was the incidence of emergence agitation (Aono’s scores ≥2) after general anesthesia. The recovery time and NRS score were assessed as secondary endpoints. All study variables were evaluated and recorded by an investigator who was blinded to the patients’ group allocation.

SPSS software version 22 (SPSS Inc.; Chicago, IL, USA) was used for the statistical analyses and all values are expressed as the number of patients (proportion), mean (standard deviation [SD]), or the median (interquartile range [IQR] or confidence interval [CI]) as appropriate. The sample size was calculated based on the primary endpoint of agiatation incidence. On the basis of an institutional preliminary study, we determined that 45 patients would be required in each group to detect a 25% difference in the incidence of Aono’s scores ≥2 between the groups, with a power of 80% and an alpha level of 0.05. Allowing for a 20% drop-out rate during the study period, we enrolled 54 patients in each group. Two-way repeated-measures analysis of variance was used for data on blood pressure, heart rate, oxygen saturation, and BIS. The Student *t-*test was used for the comparisons of intragroup values of intraoperative and postoperative mean blood pressure and heart rate. Nonparametric data such as NRS and Aono’s four-point scale were compared between groups with the Mann-Whitney *U* test. The Fischer exact test was used to compare patient sex; the percentage of patients in each group with intraoperative movement; the number of patients rescued with meperidine or intravenous labetalol or ephedrine; and those with episodes of severe EA. A *P-*value of 0.05 or less was considered statistically significant.

## Results

All study participants underwent successful CR of the nasal bone fracture without any complications associated with anesthesia or surgery; the data of 90 of the 108 eligible participants between October 2012 and September 2013 were used in the analysis (Fig. 1). Patient characteristics and operation-related data, including the operation duration and the use of nasal packing after surgery, were not statistically different between the groups (Table 1).

**Table 1:** Patient characteristics and operation-related data for each group

	Control (*n* = 45)	Dexmedetomidine (*n* = 45)
Age, years	39 (15)	36 (12)
Male	31 (69)	34 (76)
Weight, kg	67 (11)	69 (13)
BMI, kg‧m^−2^	23.4 (2.8)	23.3 (3.4)
Operation time, min	6 (5)	6 (4)
Postoperative bilateral nasal packing	40 (89)	38 (84)

Values are number of patients (proportion) or mean (SD); *BMI* Body mass index

Blood pressure (*P* = 0.182), heart rate (*P* = 0.272) and oxygen saturation (*P* = 0.478) were similar between the two groups. The preoperative RASS scores were also comparable between the two groups (*P* = 0.073). Aono’s scores after anesthesia were lower in the dexmedetomidine group than those in the control group (median [IQR]: 1 [1] vs. 1 [1, 2], 95% CI of difference: 0.07 to 0.69, mean difference: 0.4, *P* = 0.015). Agitation duration (mean [SD]; 0.3 [1] min vs. 2.5 [6] min; mean difference: 2.1 min, 95% CI of difference: 0.37 to 3.88, *P* = 0.019) and the number of agitated patients(Aono’s score ≥ 2) were lower in the dexmedetomidine group than those in the control group (Table 2). There were also fewer patients who showed severe agitation (Aono’s score ≥ 3) in the dexmedetomidine group (Table 2). Additionally, fewer patients in the dexmedetomidine group than in the control group showed movement during the operation (number [proportion] 6 [13] vs. 0 [0], *P* = 0.01) (Table 3).

**Table 2:** Agitation scale and numeric rating scale scores

	Control (*n* = 45)	Dexmedetomidine (*n* = 45)	*P* value
NRS	4 (2–5)	3 (2–6)	0.374
Aono’s scale	1 (1–2)	1 (1–1)	0.015^a^
Agitation (Aono’s scale scores ≥2)	18 (40.0)	8 (17.8)	0.020^a^
Agitation duration (min)	2.5 (6)	0.3 (1)	0.019^a^
Aono’s scale scores ≥3	9 (20.0)	2 (4.4)	0.024^a^

Values are number (proportion), mean (SD) or median (interquartile range); NRS (numeric rating scale); ^a^, *P* value < 0.05

**Table 3:** Intra operation- and post operation-related data

	Control (*n* = 45)	Dexmedetomidine (*n* = 45)	Mean difference (95% CI)	*P* value
Pre-operative RASS scores ≤ −2	0 (0%)	2 (4%)	NA	0.49
Intra-operative movement	6 (13)	0 (0)	NA	0.01^a^
End of operation to extubation, min	5 (3)	4 (2)	1.1 (−0.4 to 2.2)	0.07
Anesthesia time, min	24 (9)	20 (6)	3.6 (0.4 to 6.7)	0.03^a^
PACU stay time, min	32 (11)	41 (11)	−9.0 (−13.9 to −4.4)	< 0.001^a^

Values are number of patients (proportion) or mean (SD); RASS: Richmond Agitation-Sedation Scale; PACU: Post anesthetic care unit; ^a^, *P* value < 0.05

There was no significant difference in the NRS pain scores between the two groups (*P* = 0.37). The duration of the PACU stay was longer in the dexmedetomidine group (mean [SD]: 32 min [11] vs. 41 min [11]: mean difference: ˗9.18, 95% CI of difference: ˗13.9 to ˗4.4, *P* < 0.001); however, the anesthesia time was shorter in the dexmedetomidine group (mean [SD]; 24 [9] min vs. 20 [6] min: mean difference: 3.6 min, 95% CI of difference: 0.4 to 6.7, *P =* 0.03) (Table 1, Table 3).

## Discussion

In this randomized, controlled trial of adult patients undergoing CR of a nasal bone fracture, pre-operative administration of intravenous dexmedetomidine demonstrated several benefits such as a lower incidence of EA, reduced agitation severity, and a shorter duration of agitation. In addition to these beneficial effects on EA, we observed more stable maintenance of intraoperative anesthesia with less movement during surgery in patients who were premedicated with dexmedetomidine. Furthermore, there was no statistical difference between the groups in terms of the incidence of complications, such as pre-operative sedation and hypo- or hypertension; however, the PACU stay duration was longer in the dexmedetomidine group.

EA after anesthesia is common during the immediate postoperative period; however, its etiology in adults is unclear, and serious sequelae of EA have rarely been studied in adult patients [14, 15]. Many factors predispose a patient to EA, which is frequently initiated by uncomfortable stimuli [16]. Nasal surgery is known to be associated with a relatively high incidence of EA [6, 13]. After a CR of a nasal fracture, external splints and nasal packing are commonly used to stabilize and protect the reduction in lieu of screw insertion or fixation. The presence of external splints and nasal packing can be uncomfortable and make it difficult for patients to breathe, possibly contributing to the development of EA. Severe EA can result in nasal bleeding, re-dislocation of the reduced fracture, and even the need for reoperation. Additionally, in patients who develop respiratory depression after anesthesia, respiratory support with a face mask or airway can be difficult.

Dexmedetomidine induces sedation and analgesia without respiratory depression [17]. Several studies have advocated the beneficial effects of intraoperative administration of dexmedetomidine for reducing the incidence of perioperative morbidities after nasal surgery, such as intraoperative bleeding, postoperative pain, and EA [13, 18, 19]. Considering the short length of CR surgery, an intraoperative infusion of dexmedetomidine as an anesthetic adjuvant may prolong the anesthesia and recovery time [20]. Previous studies have shown that a single -dose of dexmedetomidine, not as a premedication, is also effective in reducing EA and facilitating smooth extubation after pediatric adenotonsillectomy [10]. However, there were no reports about single-dose premedication of dexmedetomidine in adult patients. Thus, we aimed to evaluate the efficacy of pre-operative dexmedetomidine administration in preventing or reducing the severity of EA in adults undergoing CR of a nasal bone fracture.

In our study, fewer patients developed EA, and the severity of agitation was also significantly lower in the dexmedetomidine group as compared to previous studies. Two patients in the dexmedetomidine group developed pre-operative sedation (RASS score ≤ − 3, no verbal response); however, no patients showed desaturation (SpO_2_ < 95%), and the incidence of sedation was not statistically different between the two groups (*P* = 0.49). Even though the duration of the PACU stay was significantly longer in the dexmedetomidine group than in the control group, the anesthesia time was significantly shorter in the dexmedetomidine group. This may be due to a higher incidence of agitation in the control group. Transport to the PACU after extubation is sometimes delayed in patients who experience EA because of patient safety concerns. In this study, the time from the end of surgery to extubation was not significantly different between the two groups, and the duration of the PACU stay was clinically acceptable in both groups (32 min vs. 41 min).

Dexmedetomidine can cause hemodynamic changes including hypotension, hypertension, and bradycardia [21]. We analyzed the patients’ mean arterial pressure at three-time points (baseline, pre-operative, and postoperative in the PACU) and there were no significant differences between the groups (*P* = 0.75). Furthermore, there was no patient who showed hypotension that required ephedrine in both groups. One patient developed bradycardia (heart rate < 45 beats per minute) and was treated with ephedrine; however, the incidence of bradycardia was not statistically different between the groups (*P* = 0.93). The use of pre-operative dexmedetomidine showed no analgesic advantage as the NRS pain scores in the PACU were not different between the two groups. Even though a standardized anesthetic technique and administration of a muscle relaxant was used in both groups, significantly fewer patients in the dexmedetomidine group showed movement during the operation; therefore, pre-operative dexmedetomidine may be useful as an adjuvant anesthetic to help maintain stable intra-operative anesthesia.

## Limitations

First, we did not assess the effect of different doses of dexmedetomidine. According to the results of a previous study, a single dose of dexmedetomidine (0.5 μg•kg^− 1^) was effective in reducing EA in children [10]. In this study, we used a larger dose of 1 μg•kg^− 1^, and there was no significant difference in the incidence of hypo- or hypertension, bradycardia, or other side effects related to dexmedetomidine between the two groups. Second, we did not evaluate the effect of dexmedetomidine on the inhalational anesthetic dose. The dose of sevoflurane was titrated to maintain a specific BIS range, but we did not compare the inhalational anesthetic consumption and we could not directly assess the anesthetic-sparing-effect of the premedicated dexmedetomidine. It would be worthwhile to perform a future study using a different dose of dexmedetomidine and controlled inhalational anesthetic drug.

## Conclusions

This study concluded that the administration of pre-operative dexmedetomidine lowers the incidence of EA, reduces agitation severity, and shortens the duration of agitation without complications after CR of a nasal bone fracture. The administration of pre-operative dexmedetomidine can also minimize patient movement during the operation.

## Data Availability

The datasets generated and analyzed during the current study are not publicly available as permission from participants to publicity share the dataset has not been obtained. However, these datasets are available from the corresponding author on reasonable request.

## Appendix

**Table 4:** Aono’s four-point scale

Calm	1
Not calm but could be easily consoled	2
Moderately agitated or restless and not easily calmed	3
Combative, excited, or disoriented, thrashing around	4

## Abbreviations

BIS: Bispectral index
CI: Confidence interval
CR: Closed reduction
EA: Emergence agitation
IQR: Interquartile range
NRS: Numeric rating scale
PACU: Post-anesthesia care unit
RASS: Richmond Agitation-Sedation Scale score
SD: Standard deviation
